# Stroke-Like Symptoms Status-Post Tenecteplase (TNK) Administration: A Rare Case of Hemiplegic Migraine

**DOI:** 10.7759/cureus.81850

**Published:** 2025-04-07

**Authors:** Sondos Badran, Hibah Khan, Renard Jerome, Vivian Tieu, Sydney Townsend, Johnny Randhawa, Niki Mohammadi

**Affiliations:** 1 Internal Medicine, Arrowhead Regional Medical Center, Colton, USA; 2 Internal Medicine, California University of Science and Medicine, Colton, USA

**Keywords:** functional neurological disorder, hemiplegic migraine, internal med, stroke, tenecteplase (tnk)

## Abstract

Hemiplegic migraine (HM) is an uncommon type of migraine, often misdiagnosed as an ischemic stroke due to its similar clinical presentation. We present a case of a 39-year-old female, with a past medical history of migraine headaches, who presented to the emergency department with sudden onset left-sided facial droop and left lower extremity deficits for two hours. A CT scan of the head was negative for any acute intracranial hemorrhage. The National Institutes of Health Stroke Scale (NIHSS) score was 5, and tenecteplase (TNK) was subsequently administered. Further work-up revealed unremarkable MRI of the brain with and without contrast, sinus rhythm serial EKGs without evidence of any arrhythmias, and unremarkable troponins. The transthoracic echocardiogram (TTE) was unremarkable for any intracardiac shunts. The patient’s symptoms were ultimately attributed to HM, given that the work-up for all other etiologies was ruled out. It is crucial for clinicians to perform thorough histories and physical exams for prompt detection and management of HMs and to minimize exposure to the potential adverse effects of thrombolytic agents.

## Introduction

Although migraines are a common condition affecting 15-20% of the general population, hemiplegic migraines (HM) are a rare condition with a reported prevalence of 0.01% [1]. HM is most commonly characterized by fully reversible unilateral motor weakness but can also be accompanied by other auras (e.g., visual defects, ataxia, or aphasias). The duration of symptoms is generally transient, lasting from a couple of hours to days, but in some cases, complete recovery can take weeks [2]. HM may occur sporadically or due to familial inheritance. According to Jen JC (2021), 7-15% of familial HM cases may be attributed to genetic mutations involving ion transport, notably the CACNA1A, ATP1A2, and SCN1A genes [3].

Given that hemiplegia is the defining characteristic of HM, its presentation can be challenging to assess in the emergent setting because it mimics a plethora of neurological pathologies, most commonly ischemic strokes. Identifying the correct etiology for a patient’s presentation is paramount because timing is crucial when it comes to the preservation of neuronal integrity.

Ideally, thrombolytics, such as alteplase and tenecteplase (TNK), are administered within 4.5 hours of symptom onset to be effective and prevent permanent sensorimotor deficits [4]. These medications are not benign and are associated with bleeding complications, anaphylaxis, thromboembolism, and arrhythmias [5-6]. Hence, the importance of obtaining a thorough patient history and physical exam, and utilizing clinical tools to confirm the patient’s diagnosis in the emergent setting, are crucial.

## Case presentation

A 39-year-old female with a past medical history of migraines without auras, carpal tunnel syndrome status post left carpal tunnel release surgery with nerve repair and graft five months ago, and major depressive disorder (MDD) presented to the hospital with a complaint of sudden onset of left-sided facial droop, left upper extremity deficits with worsening of baseline left hand paresthesia, and left lower extremity deficits. The patient stated that her symptoms began approximately 2-3 hours prior to arrival at the hospital with associated symptoms of a persistent right-sided occipital headache for the past two days, rating it an 8/10 in severity, and without any relief from over-the-counter ibuprofen. She reported that since her surgery five months ago, she has had residual left hand weakness and paresthesia, but the paresthesia acutely worsened 2-3 hours ago.

Upon arrival, the National Institutes of Health Stroke Scale (NIHSS) was 5 and Glasgow Coma Scale (GCS) was 15. Code stroke was activated. CT scan of the head without IV contrast, CT perfusion studies, and CTA of the head and neck were all unremarkable for any acute findings. Vital signs and initial labs on admission were all within normal limits (Table 1). Tele-Neurology was consulted and given that the patient was within the window to receive thrombolysis and without any contraindications, TNK was administered. The patient was then admitted to the Neurological Intensive Care Unit (Neuro-ICU) overnight for hourly neurologic exams.

**Table 1:** Laboratory values on admission. CO_^2^_: Carbon Dioxide; BUN: Blood Urea Nitrogen; HDL: High-Density Lipoprotein; LDL: Low-Density Lipoprotein; TSH: Thyroid-Stimulating Hormone; Ab: Antibody: IGG: Immunoglobulin G; IGM: Immunoglobulin M.

Laboratory Study	Reference Values	Measured Values
WBCs	4.5-11.1 x10^3^/μL	8.3 x10^3^/μL
RBCs	4.50-5.90 x 10^6^/μL	4.95 x 10^6^/μL
Hemoglobin	13.0-17.0 g/dL	13.7 g/dL
Hematocrit	41-53%	41%
Platelets	120-360 x10^3^/μL	257 x10^3^/μL
Sodium	135-148 mmol/L	137 mmol/L
Potassium	3.5-5.5 mmol/L	4.0 mmol/L
Chloride	98-110 mmol/L	103 mmol/L
CO2	24-34 mmol/L	24 mmol/L
BUN	8-20 mg/dL	10 mg/dL
Creatinine	0.50-1.50 mg/dL	0.60 mg/dL
Glucose	65-125 mg/dL	99 mg/dL
Calcium	8.5-10.5 mg/dL	10 mg/dL
Phosphorus	2.4-4.4 mg/dL	3.0 mg/dL
Magnesium	1.6-2.3 mg/dL	2.0 mg/dL
Troponin I	0.00-0.30 ng/mL	<0.30 ng/mL
Cholesterol	<200 mg/dL	173 mg/dL
Triglycerides	≤150 mg/dL	97 mg/dL
HDL	>40 mg/dL	46 mg/dL
LDL	<100 mg/dL	113 mg/dL
TSH	0.35-5.5 mIU/L	3.35 mIU/L
HIV-1/HIV-2 Ab	Non-Reactive	Non-reactive
Syphilis IGG/IGM Ab	Non-Reactive	Non-reactive

**Figure 1:**
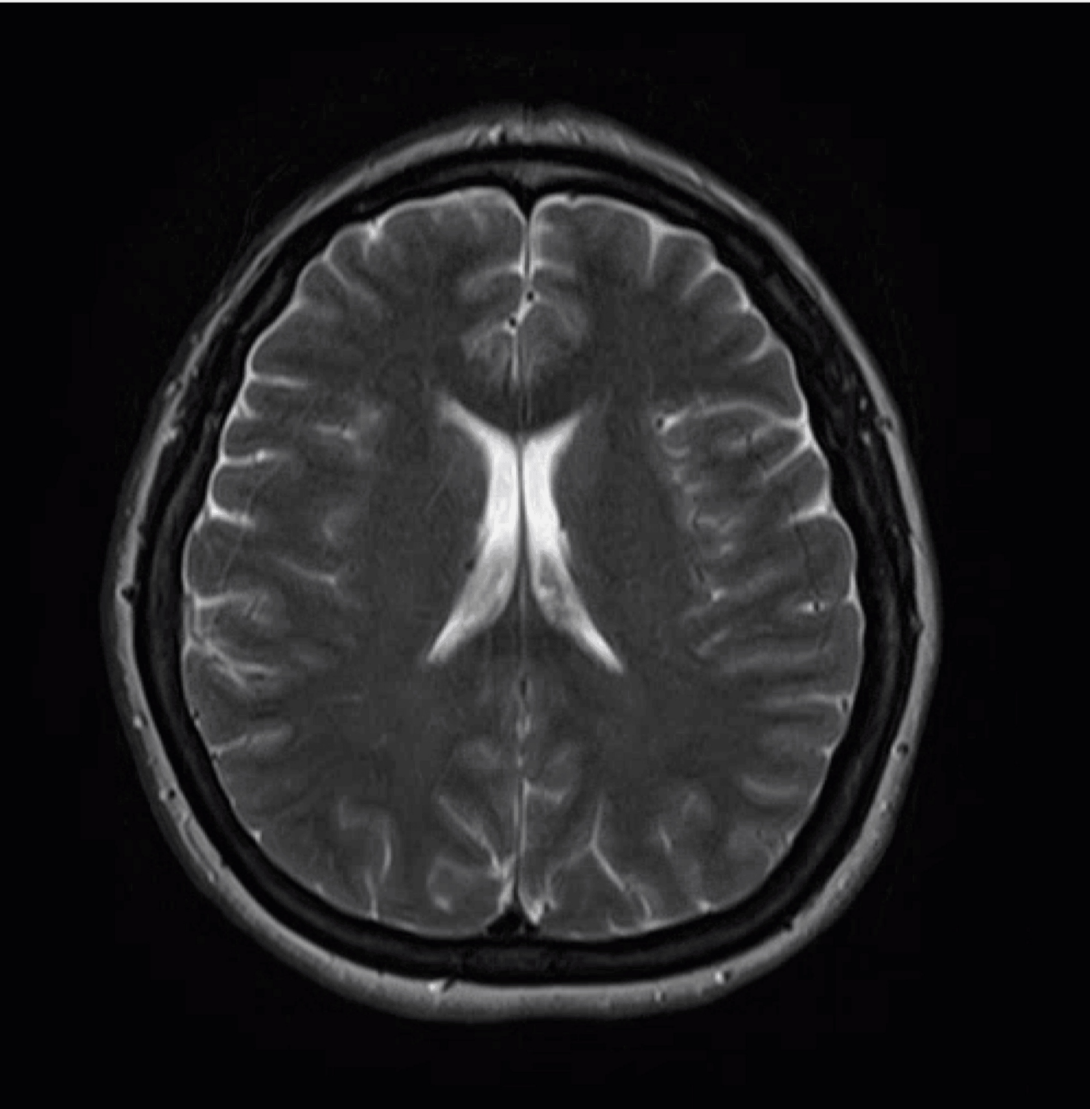
MRI brain without contrast in T2W axial view showing a normal MRI with no evidence of focal diffusion restriction to suggest acute or subacute infarction, masses, midline shift, or hemorrhages. T2W: T2-Weighted.

The next morning, the patient’s symptoms, including her sensory and motor deficits, had improved and her chronic residual left hand paresthesia had returned to baseline, although the right occipital headache persisted. Physical exam was remarkable for reduced sensation to temperature in the left V1, V2, and V3 branches. The patient had a left-sided downward drift without pronation; right upper extremity and right lower extremity were 4 out of 5 muscle strength, and left upper extremity and lower extremity 5 out of 5. The patient’s response to vibration was midline splitting over the glabella and sternum. An MRI of the brain without contrast was performed and was unremarkable for any intracranial pathology (Figure 1). Serial EKG and troponins were negative, and a transthoracic echocardiogram (TTE) with bubble study was negative for any intracardiac shunts. Given these findings, the patient’s symptoms were determined to be unlikely due to an ischemic cause and more likely due to hemiplegic migraine versus functional neurological disorder (FND). Neurology was consulted. Due to the patient's history of recurrent headaches, hemiplegic migraines were their top differential.

The patient was initially treated with IV acetaminophen and ketorolac with some improvement in her headache. However, due to persistence, she received a one-time dose of abortive therapy with sumatriptan 50 mg orally and was started on topiramate 25 mg daily for migraine prophylaxis. Her headache and symptoms resolved the day after starting these medications, and she was discharged home with plans to follow up with her primary care provider and Neurology outpatient.

## Discussion

Stroke mimics often occur in younger patients, diagnosed in one out of five patients [7]. Some stroke mimics include seizures, complex migraines, functional neurological disorder, and demyelinating disease [7-8]. Between 2% and 15% of patients presenting with symptoms of stroke mimics receive thrombolysis [7]. Jacobsen E et al. characterized stroke-like mimics based on the Norwegian tenecteplase stroke trial (Nor-TEST). Some demographic variables associated with stroke mimics include sensory symptoms, female gender, unemployment, absence of facial palsy, and aphasia. Migraines were found to be the most common mimic in the young population [7].

This case report outlines a middle-aged female with a history of migraine headaches who presented to the ED with stroke-like symptoms and was ultimately given TNK. This is a rare example of the 1% of patients who present to the ED for stroke evaluation that are later deemed to have their symptoms due to HM [9]. In fact, migraine with aura is the third most common stroke mimic after seizures and FND, respectively, and is the cause of 18% of inappropriate thrombolytic treatments in the emergent setting [9]. HM is a subtype of migraine with aura [1]. Another complicating factor is that having a history of migraine with aura increases the risk of stroke itself [1], in addition to common risk factors such as hypertension, diabetes, and tobacco use. Thus, it can be challenging to determine if an individual’s symptoms are due to a non-ischemic versus ischemic etiology.

FND was part of the neurologist’s differential diagnosis, which is a diagnosis of exclusion. Patients can present with a positive Hoover sign, drift without pronation, and global weakness, which have a high PPV [10]. Hoover’s sign has a 76-100% PPV. Patients with drift without pronation have a PPV of 93-100%. Global limb weakness selectively affecting some muscle groups correlates with FND [11]. However, some key features distinguish FND from HM. Patients with FND require a multimodal approach to treatment, involving physiotherapy, psychotherapy, and psychopharmacology [12]. Patients with HM always present with unilateral weakness and, in rare cases, bilateral weakness [2]. Aura symptoms may also be seen, which include visual and sensory deficits. Unlike FND, treatment for HM may include triptans. Although triptan use is controversial in HM, a retrospective study in Finland demonstrates the safety and efficacy of using triptans in patients with HM [13]. Another retrospective study examined the adverse effects of triptans in patients with basilar and HM [14]. Notably, no ischemic vascular events, which are the primary concern when using triptans for HM, were reported. For our patient, triptans alleviated both headache and associated symptoms, supporting a diagnosis consistent with HM rather than FND.

Current literature demonstrates that a majority of patients with HM have symptoms that last from a few hours to days. However, in rare cases, it can develop acutely and last for several weeks [1], which can make differentiation from transient ischemic attack or acute ischemic stroke difficult. The frequency of migraine attacks decreases after the age of 50 as they transition into a more classical presentation without motor deficits [1]. In the ED, HMs are increasingly being correctly diagnosed [9]. In fact, there is a risk of withholding thrombolytic treatment in patients less than 50 years old with a known history of migraines compared to the same age group without a history of headaches [9].

At the same time, TNK is not a benign medication with negligible side effects. It can lead to bleeding, which can occur in any part of the body, and this risk increases in patients who are also taking anticoagulants and antiplatelets at the time of administration [5]. One of the most dangerous complications is intracranial hemorrhage due to the increased risk of mortality, with a reported incidence of 2.9% with TNK administration [5,15]. Other side effects include thromboembolic events, cholesterol embolization, and anaphylaxis [5]. Keeping these potential complications in mind, the reported rate of adverse events of TNK administration in HM patients is as low as 0.01% [9]. 

To help navigate this predicament, having a broad list of differential diagnoses is imperative because it allows for more thorough history taking, which can help elucidate other potential causes of symptoms. The time spent on history taking, however, needs to be balanced with acting in a timely manner when aiming for ischemic stroke treatment within the 4.5-hour window to prevent further detrimental outcomes [4]. The patient discussed in this case report did not develop complications with the administration of TNK. 

This case demonstrates the diagnostic dilemma physicians face when a young patient presents with a new sensorimotor deficit in the setting of migraine history. Important physical exam findings and risk factors should be assessed to determine if thrombolytics should be administered. Thrombolytic treatments increase the risk of intracranial hemorrhage and death. However, withholding treatment can lead to permanent deficits. Therefore, it is imperative to have shared decision-making when possible to reduce the incidence of thrombolytic use in patients with a low risk of ischemic stroke who are more likely to have a stroke mimic diagnosis to prevent the feared outcome of intracranial hemorrhage.

## Conclusions

HM is a rare condition that can present as a stroke mimic in the ED setting. Patients with this condition have an increased risk of stroke in addition to the more common vascular risk factors. It represents a diagnostic challenge because symptoms can resolve over a matter of days, yet physicians have an extremely limited time window when it comes to initiating ischemic stroke treatment. Considering that TNK has a remarkably low rate of adverse outcomes in HM cases, one can argue that the benefits greatly outweigh the risks. However, ideally, this is a compromise that should be decided through shared decision-making with the patient.
